# Ethanol-Induced Oxidative Stress Modifies Inflammation and Angiogenesis Biomarkers in Retinal Pigment Epithelial Cells (ARPE-19): Role of CYP2E1 and its Inhibition by Antioxidants

**DOI:** 10.3390/antiox9090776

**Published:** 2020-08-21

**Authors:** Natalia Martinez-Gil, Lorena Vidal-Gil, Miguel Flores-Bellver, Rosa Maisto, Javier Sancho-Pelluz, Manuel Diaz-Llopis, Jorge M. Barcia, Francisco J. Romero

**Affiliations:** 1 Department of Physiology, Genetics and Microbiology, University of Alicante, San Vicente del Raspeig, 03690 Alicante, Spain; 2 Neurobiology and Neurophysiology, School of Medicine and Dentistry, Catholic University of Valencia San Vicente Mártir, 46001 Valencia, Spain; lorena_vidal_gil@outlook.es (L.V.-G.); fj.sancho@ucv.es (J.S.-P.); jm.barcia@ucv.es (J.M.B.); 3 Doctorate School, Catholic University of Valencia San Vicente Mártir, 46001 Valencia, Spain; 4 Department of Ophthalmology, Cell Sight-Ocular Stem Cell and Regeneration Program, School of Medicine, University of Colorado, Aurora, CO 80045, USA; m.flores-bellver@ucdenver.edu; 5 Department of Experimental Medicine, Università degli studi della Campania Luigi Vanvitelli, 80138 Napoli, Italy; Rosa.MAISTO@unicampania.it; 6 Department of Surgery, School of Medicine and Dentistry, University of Valencia, 46010 Valencia, Spain; manuel.diaz@uv.es; 7 School of Health Sciences, European University of Valencia, 46010 Valencia, Spain

**Keywords:** retinal pigment epithelium, homeostasis, oxidative stress, degeneration, CYP2E1

## Abstract

The retinal pigment epithelium (RPE) plays a key role in retinal health, being essential for the protection against reactive oxygen species (ROS). Nevertheless, excessive oxidative stress can induce RPE dysfunction, promoting visual loss. Our aim is to clarify the possible implication of CYP2E1 in ethanol (EtOH)-induced oxidative stress in RPE alterations. Despite the increase in the levels of ROS, measured by fluorescence probes, the RPE cells exposed to the lowest EtOH concentrations were able to maintain cell survival, measured by the Cell Proliferation Kit II (XTT). However, EtOH-induced oxidative stress modified inflammation and angiogenesis biomarkers, analyzed by proteome array, ELISA, qPCR and Western blot. The highest EtOH concentration used stimulated a large increase in ROS levels, upregulating the cytochrome P450-2E1 (CYP2E1) and promoting cell death. The use of antioxidants such as N-acetylcysteine (NAC) and diallyl sulfide (DAS), which is also a CYP2E1 inhibitor, reverted cell death and oxidative stress, modulating also the upstream angiogenesis and inflammation regulators. Because oxidative stress plays a central role in most frequent ocular diseases, the results herein support the proposal that CYP2E1 upregulation could aggravate retinal degeneration, especially in those patients with high baseline oxidative stress levels due to their ocular pathology and should be considered as a risk factor.

## 1. Introduction

The retinal pigment epithelium (RPE) is essential for retinal health [1,2,3,4,5,6,7,8,9]. In addition to being directly involved in the vision process, RPE is the main component of the outer blood-retinal barrier and it is implicated in retinal homeostasis [4]. As a barrier, RPE restricts the access of cells and molecules from blood to neural retina and allows the exchange of nutrients and waste substances [3,4]. Another important RPE function is the release of some neurotrophic and growth factors which are part of the RPE-secretome [5] and are essential to maintaining retinal and choriocapillar function [1,3,5].

Age-related macular degeneration (AMD) is one of the major causes of blindness worldwide without cure [10]. AMD affects around 8% of the population and the prevalence of this disease is increasing as longevity does [11]. AMD is a complex and multifactorial disease where ageing plays a critical role [12]. Besides age and genetic predisposition, which have clearly been identified as risk factors, the environment and nutrition have also been described as modifiable risk factors to avoid RPE and photoreceptor cells death at eye macula [11,12]. Another significant player in AMD is the RPE, which loses its functions, promoting retinal neurodegeneration [13,14]. Choroidal angiogenesis and inflammation are the main activated pathways in AMD [15]. These processes have been related with RPE dysfunction [16,17,18,19,20] by promoting the disturbance of RPE-released growth factors such as vascular endothelium growth factor (VEGF), pigment epithelium-derived factor (PEDF) and inflammatory molecules such as interleukin (IL)-6, IL-8 [16], IL-1α and IL-1β [18] or matrix metalloproteinases (MMPs) such as MMP-1, MMP-2 and MMP-3 [20]. All this, together with the increase in oxidative stress by the accumulation of superoxide anions and other reactive oxygen species (ROS), finally causes RPE cell death. Furthermore, interaction between photoreceptors and RPE cells is critical for maintaining visual function, and its alteration can lead to compromised vision. Our group has already demonstrated that antioxidants can delay photoreceptor degeneration in a mouse model of retinitis pigmentosa [21,22,23], and this protective effect certainly requires the integrity of RPE [24,25]. 

Oxidative stress is a common factor in all retinal diseases [10,26] which contributes to inflammation and angiogenesis development in retinal neurodegenerative diseases [27,28,29]. In addition, there is a cross talk between these processes [28,30,31]. Common players such as the stress-activated protein kinases (SAPK)/Jun amino-terminal kinases (JNK), the nuclear factor kappa-B (NFkB) and protein kinase B (AKT) act as signaling factors contributing to the senescence and ageing diseases development [32]. Although RPE is able to counteract ROS by different mechanisms [33,34,35,36,37,38,39], exposure to chronic and large amounts of them promote an imbalance between its generation and elimination. Several authors have previously shown that alcohol intake increases ROS production by microsomal cytochrome P450-2E1 (CYP2E1) [40,41,42,43,44,45]. They demonstrated that CYP2E1-dependent ROS production can induce apoptosis and inflammation in alcohol-induced liver injury (40) and alcoholic hepatic steatosis [41,42]. Additionally, the upregulation of CYP2E1 has been related to the induction of angiogenesis in different types of cancer [43,44,45]. Even though it has been demonstrated that alcohol consumption contributes to neurological disease development and could be considered as a risk factor in AMD, the implication and activity of CYP2E1 in these neurodegenerative diseases is poorly understood [46]. 

Previous studies from our group reported that CYP2E1 is present in human RPE and its overexpression and activation promote cell death [47]. Furthermore, and based on our knowledge, few studies have been conducted in the retina and even less in the RPE. Therefore, our main goal is to study the degeneration processes activated in RPE cells after inducing oxidative stress and CYP2E1 upregulation by ethanol (EtOH) and elucidate how antioxidants could alleviate these alterations. 

## 2. Materials and Methods

### 2.1. Cell Culture and Treatments

Human RPE cell line ARPE-19 cells were cultured according to supplier’s protocol (American Type Culture Collection [ATCC]; Manassas, VA, USA) and were used from passages 11 to 13. Cells were cultured in DMEM/F12 (Thermo Fisher, Waltham, MA, USA), supplemented with 5 mM 2-[4-(2-hydroxyethyl)piperazin-1-yl] ethanesulfonic acid (HEPES; Thermo Fisher), 44 mM NaHCO_3_ (Thermo Fisher), 10% fetal bovine serum (FBS; Thermo Fisher) and 100 U/mL penicillin/streptomycin (Thermo Fisher). Cell cultures were maintained at 37 °C and 5% CO_2_. Cells were seeded at 1 × 103 cells/cm^2^ density. After 2 days at 80% of confluence, cells were treated for 24 h at different EtOH (Biosolve, Valkenswaard, The Netherlands) concentrations considering our previous results [47,48]. For CYP2E1 inhibition, diallyl sulfide (DAS; Santa Cruz Biotechnology, Dallas, TX, USA), a known CYP2E1 inhibitor, was added to the medium in a final concentration of 20 mM in 0.1% of dimethyl sulfoxide (DMSO) to the culture media without FBS. As an antioxidant, cells were treated with N-acetylcysteine (NAC; Sigma Aldrich, St. Louis, MO, USA) in a final concentration of 4 µM. DAS and NAC were added to the cell culture medium at the same time as EtOH and treatments were carried out for 24 h. Previously, for both drugs, a viability assay was performed to select the final concentration used (data not shown). 

### 2.2. Determination of ROS Levels

ROS levels were measured by two different fluorescent probes. We used 2-7- dichlorodihydrofluorescein diacetate (DCFDA; Santa Cruz Biotechnology) for total intracellular ROS. This molecule can be oxidized by ROS producing intracellular dichlorofluorescein (DCF), which is a fluorescent compound. Dihydroethidium, a superoxide anion indicator (DHE; Thermo Scientific, Waltham, MA, USA) exhibits blue fluorescence; however, once this probe is oxidized to ethidium, it intercalates within DNA, staining the cell nucleus with a bright fluorescent red. Cells were seeded in a 96 multiwell plate as mentioned before. After 24 h of EtOH, DAS and NAC treatment, cells were incubated with 15 µm of DCFDA and 5 µm of DHE according to supplier’s protocol, during 30 min at 37 °C, thereafter; levels of fluorescence were measured with a multiplate reader (Victor X5, Perkin Elmer, Turku, Finland). The experiments were repeated in three different days (three independent experiments, N = 3) for DHE incubation and four times (N = 4) for DCFDA to ensure the consistency of the results. The results were expressed as percentage relative to the control group. 

### 2.3. Cell Viability

The cell proliferation kit II (Roche, Basel, Switzerland) based on the cleavage of the yellow tetra-zolium salt sodium 3′-[1-(phenylaminocarbonyl)-3,4-tetrazolium]-bis (4-methoxy-6-nitro) benzene sulfonic acid hydrate (XTT), to form an orange formazan dye, was used to determine cell viability as mitochondrial activity. Cells were seeded in a 96-well plate and treated as mentioned before in 2.1 cell culture and treatments section. According to supplier’s protocol, XTT solution was added to each well and incubated for 6 h at 37 °C in 5% CO_2_. Then, absorbance was read at 550 nm by microplate reader (Victor X5; Perkin Elmer). The experiments were repeated in three different days (three independent experiments, N = 3) to ensure the consistency of the results. The results were expressed as percentage relative to the control group.

### 2.4. Proteome Profiling

The human angiogenesis proteome profile array (R&D Systems, Minneapolis, MN, USA) provides a rapid, sensitive tool to simultaneously detect the relative levels of angiogenesis- and inflammation-related proteins in a single sample. For protein isolation, EtOH-treated ARPE-19 cells were rinsed in phosphate-buffered saline (PBS) and lysed in RIPA lysis buffer (Thermo Fisher Scientific) supplemented with a protease/phosphatase inhibitor cocktail (Sigma-Aldrich). Subsequently, the samples were sonicated 3 cycles of 3 pulses (waiting 10 s between pulses) at 20% of amplitude, (Branson Digital Sonifier) and centrifugated at 100,000× *g* at 4 °C for 20 min. The amount of protein in supernatants was quantified by BCA Protein Assay (Thermo Fisher Scientific) using bovine serum albumin as standard. ARPE-19 cells were exposed to different EtOH concentrations in triplicate. Cells from the same experimental condition were pooled before protein extraction. After that, a total of 200 µg of proteins from each pool of samples was incubated in the immunoblotted membranes overnight at 4 °C, according to the manufacturer’s manual. The day after, each membrane was incubated for 30 min at room temperature with streptavidin-HRP secondary antibody. The chemiluminescence signal was detected by CCD camera (ImageQuant LAS 4000 Mini, GE, Chicago, IL, USA). Signal intensity was quantified by densitometry using the ImageQuant TL (GE) software and was determined by the average signal of the pair of duplicate spots representing each protein. The row data of the quantification are available in the Supplementary Material (Table S1).

### 2.5. Matrix Metalloproteinases ELISA

The quantitative determination of matrix metalloproteinases (MMP-1, MMP-2, MMP-3, MMP-7, MMP-8, MMP-9 and MMP-13) was carried out by an ELISA kit, Mosaic ™ ELISA Human MMP Panel (R&D Systems) according to the manufacturer’s protocol. First, using the same procedure described before for proteome profiling, proteins were isolated. Then, a total of 100 µL of each protein sample was deposited in the ELISA plate well containing fixed specific capture antibodies for each MMP. Chemiluminescent signal was detected by CCD camera (ImageQuant LAS 4000 Mini, GE). Signal intensity was quantified by densitometry using the ImageQuant TL (GE) software. MMPs’ concentration values were calculated using each standard curve and were normalized considering the protein concentration of each sample. The experiments were repeated on three different days (three independent experiments, N = 3). The results were expressed as percentage relative to the control group.

### 2.6. Western Blotting

After EtOH treatment, cells were scraped and lysed with RIPA buffer as previously described in 2.4. Proteome profiling section. Thereafter, protein quantification was carried out by BCA Protein Assay (Thermo Fisher Scientific) using bovine serum albumin as standard. Equal amounts of protein from each sample (35 μg) were denatured in Laemmli sample buffer (4% SDS (*w/v*), 10% (*v/v*) beta-mercaeptoethanol, 20% (*v/v*) glycerol, 0.004% (*w/v*) bromophenol blue and 125 mM Tris-HCl, pH 6.8) and heated to 95 °C for 10 min. Then, electrophoresis was carried out by SDS-PAGE on 4–12% (*v/v*) acrylamide gels and electroblotted onto polyvinylidene difluoride membranes (PVDF; Millipore, Temecula, CA, USA). Membranes were incubated overnight at 4 °C with rabbit anti-vascular endothelial growth factor receptors 1 and 2 (VEGFR-1 and VEGFR-2) antibodies (1:250; Abcam, Cambridge, MA, USA), rabbit anti-P65-NFkB antibody (1:500; Santa Cruz Biotechnology), rabbit anti-CYP2E1 antibody (1:250; Abcam), rabbit anti-SAPK/JNK antibody (1:250; Cell Signaling Technology, Danvers, MA, USA), rabbit anti-AKT antibody (1:500; Cell Signaling Technology), rabbit anti- phospho (Ser473)-AKT antibody (1:250; Cell Signaling Technology) and mouse anti-β-actin antibody (1:500; Santa Cruz Biotechnology) diluted in 3% (*w/v*) bovine serum albumin in Tris-buffered saline with 0.1% Tween-20 (TBS/T) at pH 7.6. Finally, membranes were incubated for 2 h at room temperature with anti-rabbit or anti- mouse IgG-HRP (1:10,000; Santa Cruz Biotechnology) diluted in TBS/T after three 10-min washes with TBS/T. Bands were visualized with enhanced chemiluminescence (ECL; Pierce, Thermo Fisher Scientific) and detected with Image Quant LAS-4000 mini (GE). Protein levels were quantified by densitometry using ImageJ software (National Institutes of Health [NIH], Bethesda, MD, USA). The results were normalized by β-actin values as a housekeeping protein. The experiments were repeated in three different days (three independent experiments, N = 3) to ensure the consistency of the results. The results were expressed as percentage relative to the control group. 

### 2.7. Quantitative Real Time PCR (RT-qPCR)

Total RNA was extracted from ARPE cells after treatments by RNeasy Plus Micro/Mini Kits (Qiagen, Hilden, Germany) and Nanodrop (Thermo Fisher Scientific) was used to evaluate the total RNA concentration and its quality (260/280 absorbance ratio). For each reaction, 100  ng RNA was reverse-transcribed into cDNA by reverse transcription-polymerase chain reactions (RT-PCR) using SuperScript III First-Strand Synthesis System (Life Technologies, Thermo Fisher Scientific) in a thermal cycler PeqSTAR 96 Universal Gradient (PeqLAb, VWR International, Amadora, Portugal) under the following reaction conditions: 65 °C for 5 minutes (min), room temperature for 2 min, 42 °C for 60 min, and 70 °C for 10 min. The mRNA expression analysis was performed using 1 µL of the cDNA synthesis reaction of each sample subjected to real-time quantitative PCR (qPCR). The reactions were performed with Sybr Green Supermix (Applied Biosystems, Carlsbad, CA, USA) in a LightCycler 480 II System (Roche) under the following reaction conditions: 95 °C for 5 min, followed by 40 cycles of 95 °C for 10 s (second), 60 °C for 20 s and 72 °C for 30 s. Samples were run in triplicate to calculate VEGF (F: 5′-AGGAGGAGGGCAGAATCATCA-3′; R: 5′-CTCGATTGGATGGCAGTAGCT-3′) and pigment epithelium derived factor (PEDF) (F: 5′-AACCTTACAGGGGCAGCCTT-3′; R: 5′-TGAGGGACACAGACACAGGG-3′) mRNA expression. The primer concentration used was 10 µM and normalization was done using the endogenous control gene GAPDH (F: 5′ TGAAGGTCGGAGTCAACGGAT-3′; R: 5′-TTCTCAGCCTTGACGGTGCCA-3′) to standardize the results. X-fold change in mRNA levels was determined by applying the 2−ΔΔCT method. The experiments were repeated in three different days (three independent experiments, N = 3). The results were expressed as percentage relative to the control group. 

### 2.8. Statistical Analysis

Statistical analyses were performed by using Prism 5.04 software (GraphPad, San Diego, CA, USA). The analysis of variance was carried out by one-way ANOVA and multiple comparisons using the post hoc Tukey test. Data are reported as the mean ± standard error of the mean. Statistically significant differences were set at *p* < 0.05.

## 3. Results

### 3.1. EtOH Induces ROS Accumulation in RPE Cells Promoting Death

**Figure 1:**
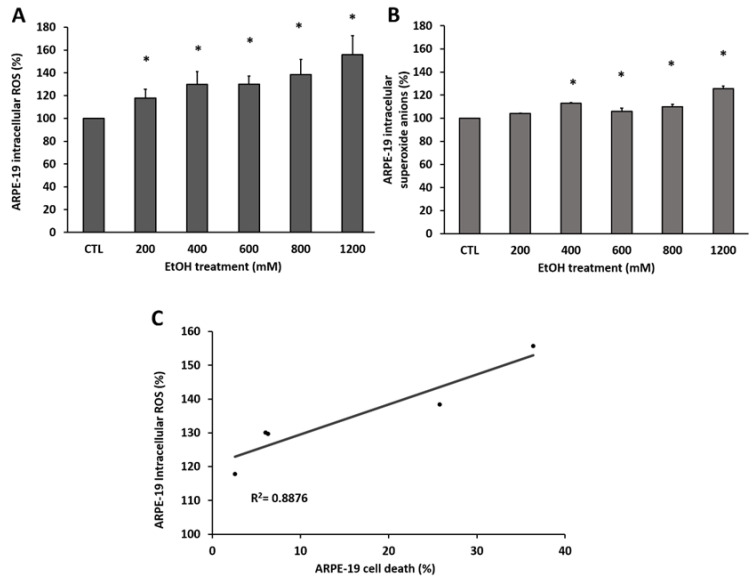
Intracellular reactive oxygen species (ROS) accumulation and cell death in ARPE-19 after ethanol (EtOH) exposure. (**A**) After 24 h of EtOH treatment with increasing concentrations, total intracellular ROS were measured by 2-7-dichlorodihydrofluorescein diacetate (DCFDA) fluorescence probe and (**B**) superoxide anions by DHE fluorescence probe. (**C**) Lineal correlation between total intracellular ROS and cell death measured by XTT. Values are expressed as mean ± SEM (N = 3–4). Statistically significant differences were set at * *p* < 0.05 vs. control (CTL) group.

Previously published works from our group showed that RPE cells are very resistant to EtOH-induced cytotoxicity and more than 600 mM of EtOH is necessary to induce cell death by apoptosis. For this reason, and considering our preliminary data, our first objective was to measure EtOH-induced ROS accumulation in ARPE-19 cells. Two fluorescence probes were used to determine EtOH-induced ROS in human RPE cells. The DHE probe was selected to measure superoxide anions and DCFDA to detect total of intracellular ROS. ARPE-19 cells were treated for 24 h with increasing concentrations of EtOH (200, 400, 600, 800 and 1200 mM of EtOH) and the results obtained were compared with those from non-treated cells (control group). As Figure 1 shows, the total number of intracellular ROS (Figure 1A) was significantly increased in all treated groups compared to non-treated cells in a concentration-dependent manner. The increase in superoxide anions (Figure 1B) was statistically significant from 400 mM EtOH. These results were accompanied by a positive correlation (R^2^ = 0.887) between intracellular ROS accumulation and the increase in cell death, measured by cell proliferation kit II (XTT) (Figure 1C).

### 3.2. Ethanol Altered the Inflammation and Angiogenesis-Related Proteome Profile in Human RPE Cells

**Figure 2:**
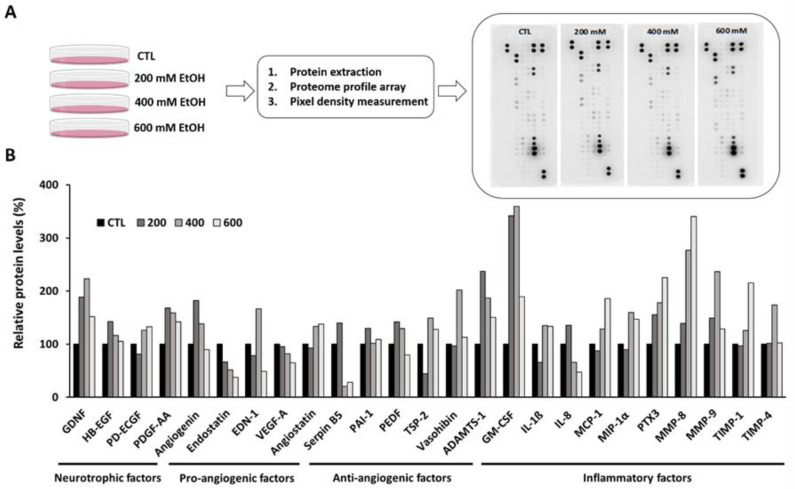
Proteome profile in ARPE-19 cells after EtOH exposure. (**A**) Workflow of the semi-quantitative proteomic analysis by antibody array system after 24 h of 200, 400 and 600 mM EtOH treatment in ARPE-19 cell. (**B**) Main neurotrophic, angiogenic and inflammation factors that experienced major changes. Values are expressed as the mean of pixel density signal of the pair of duplicate spots representing each protein.

Knowing the important role of superoxide anions and ROS accumulation in oxidative stress generation, RPE cells proteome profile changes were measured starting with initial EtOH concentration in which ROS were enhanced (200 mM) until the concentration of EtOH in which cell death starts (600 mM). The use of the Proteome Profiler Human Angiogenesis Array Kit allows us to measure a wide range of molecules and growth factors released by RPE cells as a part of RPE-secretome. As Figure 2A shows, a total of 55 proteins were identified in ARPE-19 cells after EtOH treatment. In Table S1, all the raw data obtained are shown. In Figure 2B, the most relevant neurotrophic, angiogenic and inflammation factors that experienced bigger changes due to EtOH treatment are represented. Some of these factors were upregulated after EtOH exposure such as glial cell line-derived neurotrophic factor (GDNF), pro-angiogenic factors VEGF-C and granulocyte macrophage colony-stimulating factor (GM-CSF) and MMP-8 as inflammation-related factors. Nevertheless, other pro-angiogenic factors such as VEGF-A or endostatin were downregulated. This proteome analysis also showed that there is a biphasic response in some released factors. Note that PEDF, an anti-angiogenic factor, experienced an increase at 200 mM EtOH followed by a drop in its protein levels. Among others, the inflammation-related factors IL-8, MMP-9 and metalloproteinase inhibitor-4 (TIMP-4) showed similar behavior. 

### 3.3. Ethanol Modified Matrix Metalloproteinases Levels in Human RPE Cells

**Figure 3:**
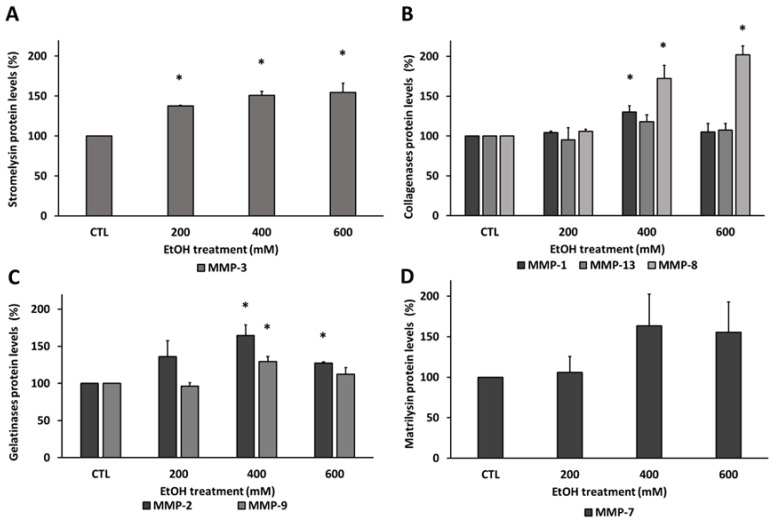
Metalloproteinase (MMP) protein levels in ARPE-19 cells under EtOH treatment. The levels of MMPs were measured with ELISA. The relative MMPs protein levels after EtOH treatment were represented by their functional classification: (**A**) stromelysin, (**B**) collagenases, (**C**) gelatinases and (**D**) matrilysin. Values are expressed as mean ± SEM (N = 3). Statistically significant differences were set at * *p* < 0.05 vs. CTL group.

To validate the proteome array and because MMPs have been considered an important angiogenesis and inflammation biomarkers in AMD pathogenesis, MMPs were quantified by ELISA after EtOH exposure. The results in Figure 3 show a graphical representation where MMPs are grouped according to their cellular function. According to ELISA results, EtOH promoted a significant change on MMPs protein levels in RPE cells. While some MMPs were significantly upregulated in all EtOH concentration used, others only experienced this alteration at the highest concentrations used. Interestingly, stromelysin MMP-3 was enhanced (Figure 3A) in all EtOH concentrations used showing upregulation in a concentration-dependent manner. Among collagenases (Figure 3B), MMP-8 showed a marked increase at 400 mM EtOH which reaches around 200% at 600 mM EtOH compared with control group. On the other hand, the MMP-1 differences were only observed at 400 mM EtOH compared with the control group. No significant differences were observed in MMP-13 protein levels. The gelatinases family experienced an increment at 400 mM EtOH compared to non-treated group (Figure 3C), these differences being greater in MMP-2. Interestingly, both MMP-2 and MMP-9 were decreased at 600 mM EtOH, returning their protein values to the lowest EtOH concentration used. Finally, there were not significant differences in the matrilysin MMP-7 (Figure 3D). 

### 3.4. Ethanol Modified the Upstream and Downstream Angiogenesis Regulators in RPE Cells 

**Figure 4:**
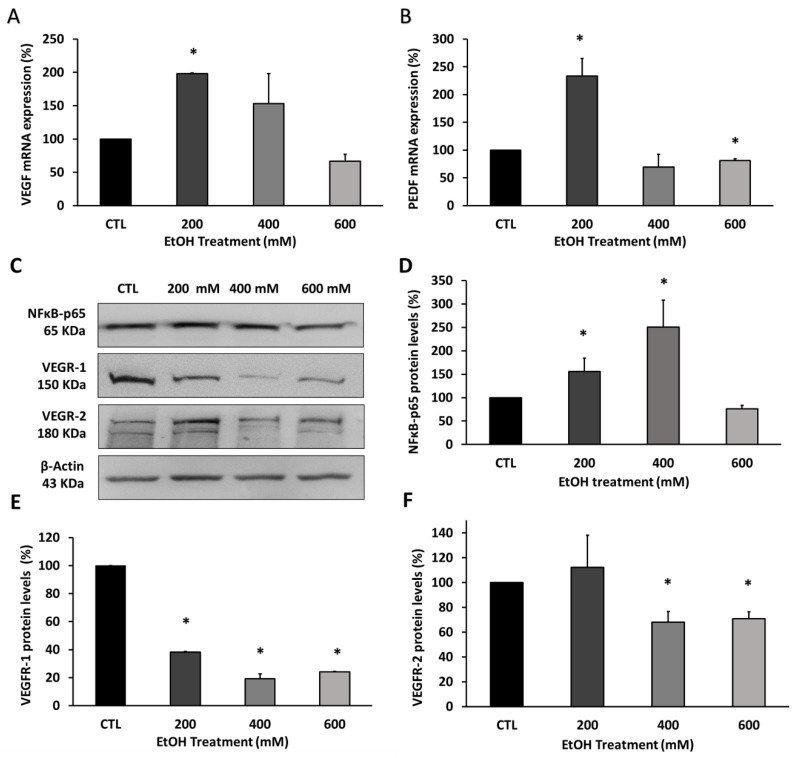
Growth factors and angiogenesis biomarkers in ARPE-19 cells after EtOH treatment. (**A**) VEGF and (**B**) PEDF mRNA expression quantification by qPCR. (**C**) Representative pictures of protein levels quantification by western blot (WB) of (**D**) p65-NFкB, (**E**) VEGFR-1 and (**F**) VEGFR-2. Gene expression was normalized by GAPDH gene expression. Protein levels were normalized by β-actin. Values are expressed as mean ± SEM (N = 3). Statistically significant differences were set at * *p* < 0.05 vs. CTL group.

In order to confirm the effect of EtOH in the release of the main angiogenesis factors by RPE and validate the proteome array, the expression and translation of upstream angiogenesis regulators were quantified (Figure 4). VEGF and PEDF mRNA expression were studied by qPCR in ARPE-19 cells after EtOH treatment at different concentrations. 

The lowest EtOH concentration used, 200 mM EtOH, was enough to overexpress VEGF and PEDF. The VEGF mRNA reached twice the level at 200 mM EtOH compared with the control group (Figure 4A). Similarly, PEDF mRNA was overexpressed by around 150% at 200 mM EtOH (Figure 4B). However, the increment of EtOH concentrations resulted in a decrease in VEGF and PEDF expression, being statistically significant in the case of PEDF. These results are in line with those obtained in the proteome profile array and correlate with MMP protein levels, which also experienced a biphasic response depending on EtOH concentration used. To clarify if EtOH also modified the upstream and downstream angiogenesis regulators in human RPE cells, protein levels of the VEGF transcription factor NFkB-p65 and the VEGF receptors (VEGFR-1 and VEGFR-2) were measured by Western blot, (Figure 4C). Similarly to what happened with VEGF, while treatment with 400 mM EtOH significantly increased NFkB-p65 protein levels, 600 mM EtOH had the opposite effect, even falling below baseline levels, (Figure 4C,D). On the other hand, there is a significant decrease in an EtOH concentration-dependent manner of VEGFR-1 (Figure 4C,E). The VEGFR-2 protein expression was increased at 200 mM EtOH followed by a significant reduction at 400 and 600 mM EtOH (Figure 4C,F).

### 3.5. CYP2E1 Upregulation Promotes Cell Death via Oxidative Stress Induction

**Figure 5:**
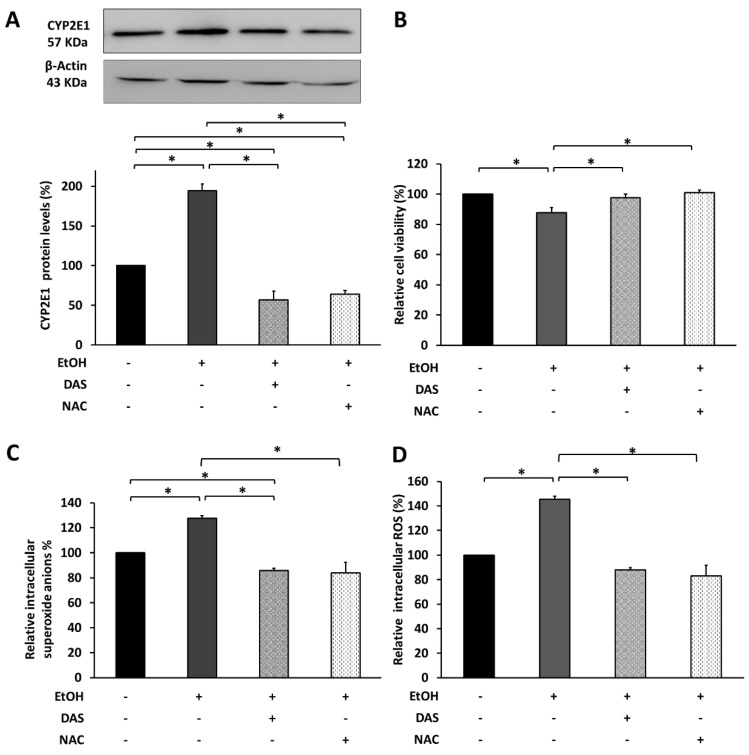
EtOH-induced oxidative stress promotes CYP2E1 upregulation and cell death. (**A**) CYP2E1 protein levels in ARPE-19 after 600 mM EtOH treatment and 20 mM DAS or 4 μM NAC measured by WB. (**B**) Cell viability by XTT assay. (**C**) Superoxide anions by DHE fluorescence and (**D**) Total intracellular ROS by DCFH fluorescence. Protein levels were normalized by β-Actin. Values are expressed as mean ± SEM (N = 3). Statistically significant differences were set at * *p* < 0.05.

Based on previously published results from our group, 600 mM EtOH is necessary to upregulate CYP2E1. With the aim to elucidate the implication of CYP2E1 in EtOH-induced oxidative stress, ARPE-19 cells were treated at the same time with 600 mM EtOH and 20 mM DAS (CYP21E1 inhibitor) or 4 µM NAC (antioxidant molecule). As Figure 5 shows, both drugs were able to reduce CYP2E1 protein levels to control values (Figure 5A). The protective effect of CYP2E1 inhibition and ROS blockade resulted in an increase in cell survival, reaching the values of the control group (Figure 5B). This improvement of cell survival correlates with the significant reduction in intracellular superoxide anions (Figure 5C) and total intracellular ROS (Figure 5D).

### 3.6. CYP2E1 Upregulation Modulates the Upstream Angiogenesis and Inflammation Regulators in RPE Cells

**Figure 6:**
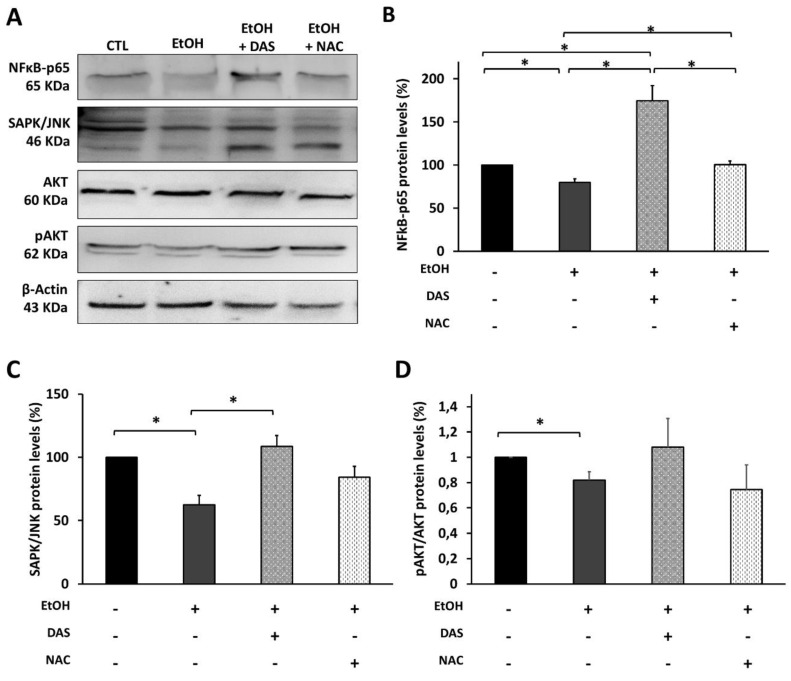
Upstream angiogenesis and inflammation biomarkers in ARPE-19 cells after EtOH treatment. (**A**) Representative pictures of protein levels quantification by WB of (**B**) p65-NFкB (**C**) SAPK/JNK and (**D**) pAKT/AKT ratio. Protein levels were normalized by β-actin. Values are expressed as mean ± SEM (N = 3). Statistically significant differences were set at * *p* < 0.05 vs.

Because 600 mM of EtOH upregulated CYP2E1 and resulted in the downregulation of angiogenesis factors, we studied the possible implication of CYP2E1 in this phenomenon. Again, ARPE-19 cells were treated simultaneously with 600 mM of EtOH and DAS to inhibit CYP2E1 or NAC to abolish ROS production (Figure 6). As expected, CYP2E1 inhibition and oxidative stress depletion increased protein levels of NFkB-p65. In addition, CYP2E1 inhibition with DAS induced an upregulation of NFkB-p65, increasing its protein levels by around 70% above the control group (Figure 6A,B). In addition, treatment with DAS promoted the increase in protein levels of the stress-activated protein kinase/c-Jun NH(2)-terminal kinase (SAPK/JNK) (Figure 6A,C) and the phosphorylated form OF protein kinase (p-AKT) (Figure 6A,D) which are involved in NFkB-p65 pathway activation. However, although treatment with NAC also increased NFkB-p65, SAPK/JNK, Pakt/AKT protein levels, it did not have the same effect as DAS.

## 4. Discussion

Previous studies from our group reported that EtOH induces cytotoxic response in human RPE cells [47,48]. While low EtOH exposure induces a pro-survival pathway activation such as autophagy [48], higher EtOH concentrations induce cell death and RPE dysfunction [47]. We also demonstrated that higher EtOH exposure induces CYP2E1 upregulation, promoting cell death through apoptosis activation [47]. However, the current work reveals that EtOH-induced oxidative stress can upregulate CYP2E1 expression and activity by itself, inducing RPE cells alterations (Figure 5). The use of NAC as an antioxidant was able to decrease CYP2E1 expression, improving cell viability in a similar way that the CYP2E1 specific inhibitor (DAS) did. Furthermore, superoxide anions and total of ROS generated by EtOH were reduced to baseline levels using both treatments. A plausible explanation is that oxidative stress generated under EtOH treatment in ARPE-19 cells is related to CYP2E1 activity, resulting in the production of large amounts of ROS [40,41,42,43,49], which were key mediators in RPE cell death (Figure 1). In accordance with Jin et al. [49], the ROS production mediated by CYP2E1 activity could be upregulating its own expression. This would be also supported by the fact that CYP2E1 is induced in ARPE-19 cells under different oxidative stress stimuli, such as H_2_O_2_ and high glucose exposure [50].

Levels of angiogenesis and inflammation-related factors were modified by EtOH treatment. While some of them were up- or down-regulated in a concentration-dependent manner, others experienced a bi-phasic response that could be explained considering survival and signaling pathways activation [47,48,51,52,53,54]. Among others, MMPs were upregulated in an EtOH concentration-dependent manner (Figure 2 and Figure 3). The expression of MMPs, which is low in healthy RPE [55], could be elevated during inflammatory, degenerative and angiogenic lesions [56]. The increase in MMP-2 and MMP-9 under EtOH treatment reinforces the claim that both are upregulated in retina under oxidative stress conditions [31,56]. EtOH treatment also increased MMP-1 and MMP-3; both have been involved in extracellular matrix disruption surrounding the RPE and the Bruch’s membrane [20,31,57]. The increase in MMP-8 also corroborates the activation of the inflammation process in RPE under EtOH exposure [58]. 

It has been demonstrated that RPE releases pro-angiogenic factors such as VEGF, which stimulates the proliferation of endothelial cells and is also implicated in the development of pathophysiology conditions in AMD [3,59]. Thus, the use of VEGF as a target of therapeutic strategies has been vastly explored [60]. VEGF, mainly secreted into the basal side of the RPE retinal layer, is implicated in cell survival and vascular maintenance. The increase in VEGF in ARPE-19 cells at 200 mM EtOH (Figure 4) could be explained by its implication in cell survival pathways under oxidative stress conditions [47,48,51]. However, the large amounts of ROS produced at 600 mM EtOH have the opposite effect. A plausible explanation could be that greater amounts of ROS induce CYP2E1 upregulation, enhancing oxidative stress and promoting cell death by apoptosis [47,61]. CYP2E1 upregulation could also act by blocking the VEGF pathway not only in RPE but also in retinal vasculature [50,51], which would result in a dysfunction of the outer blood–retinal barrier. Our present data also show an increase in PEDF at 200 mM EtOH, followed by a significant fall in a dose-dependent manner (Figure 4). PEDF is one of the major regulators of retinal angiogenesis [53], being able to inhibit the VEGF effect by binding to VEGFR-2 or by promoting the proteolysis of VEGFR-2 by the activation of α-secretase [62]. Therefore, our results could indicate that RPE cells compensate for the VEGF overexpression through the increase in PEDF. Moreover, PEDF could be helping to counteract ROS-induced cell death, considering its neurotropic and anti-inflammatory activity [54,63]. The fact that the induction of VEGF promotes VEGFR-2 upregulation [51] allows us to explain our results. As it is possible to see in Figure 4, protein levels of VEGFR-2 have the same response to EtOH exposure that VEGF had. In addition, NFkB-p65 showed a similar behavior which is in agreement with these results, considering that constitutive VEGF secretion in the RPE/choroid is regulated by this transcription factor [17]. In addition, NFkB-p65 has been found to be upregulated under angiogenic [14] and inflammatory conditions [64]. 

There are many reports that suggested a possible relationship between CYP2E1 and NFkB, but the roles played by each of them remain unclear. While some authors defend the idea that CYP2E1 plays a fundamental role in the regulation of NFkB [41,65], others affirm that NFkB is the main mediator in CYP2E1 expression [66]. Considering our results, the inhibition of CYP2E1 by DAS promoted an increase in NFkB-p65 protein levels, corroborating the relationship between both (Figure 6). Furthermore, the fact that NFkB-p65 not only was upregulated by the CYP2E1 inhibition, but also its protein levels increased above those of the control group, potentially indicating a possible relationship between CYP2E1 in angiogenesis and inflammation upstream regulators in human RPE cells, in a similar way as it does in other tissues [40,41,42,43,44,45,46]. Anyhow, further studies are needed to clarify this relationship in RPE. The inhibition of CYP2E1 also modified the pAKT/AKT protein profile, which is also implicated in angiogenesis signaling mediated by VEGF/VEGFR-2 [67], although these changes were not statistically significant. Importantly, SAPK/JNK, which was decreased after EtOH exposure, experienced an increase after NAC and DAS treatment. This could be explained considering results from Cao et al. [68], which attributed to JNK an antiapoptotic role. Additionally, JNK has been suggested as a neovascularization modulator, acting as a VEGF transcription factor [69]. This would correlate with the increased protein levels of NFkB-p65 also observed in Figure 6, which has been demonstrated to be involved in the JNK signaling pathway [70]. 

**Figure 7:**
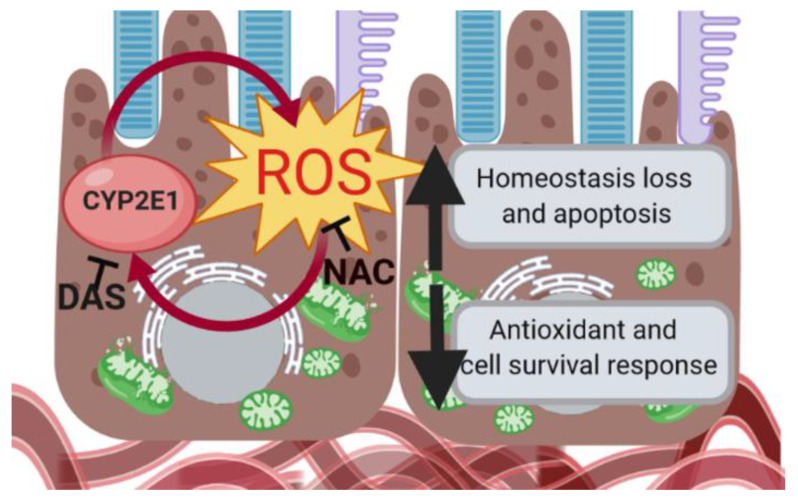
CYP2E1 induces oxidative stress in retinal pigment epithelium (RPE) cells promoting outer-blood retinal barrier degeneration. Low levels of ROS in RPE cells activates their antioxidant response and cell survival pathways. In contrast, the activation of CYP2E1 by accumulation of ROS induces its own regulation, increasing oxidative stress which results in cell death and finally RPE dysfunction. Created by BioRender.com.

Inflammation and angiogenesis are the main processes activated during retinal neurodegenerative diseases [17], including AMD [13,30,71], one of the major causes of blindness worldwide without cure [10] in which RPE plays an important role. The implication of CYP2E1 in RPE dysfunction and its role in outer blood–retinal barrier degeneration has not been previously studied. Thus, the fact that levels of angiogenesis- and inflammation-related factors were modified after EtOH-induced oxidative stress in RPE cells, could indicate that CYP2E1 upregulation affects RPE function. Although CYP2E1 would play a key role in the generation of ROS and also in the alteration of the angiogenesis and inflammatory proteome profile in RPE cells, further studies are needed to understand the role of CYP2E1 in retinal diseases and specifically in outer blood–retinal barrier dysfunction. Herein, our results demonstrate that human RPE cells have different responses depending on their baseline oxidative stress levels. At high oxidative stress levels, the induction of CYP2E1 expression and activity not only by EtOH but also by ROS, would be activating cell death and RPE degeneration promoting the progression of retinal diseases (Figure 7). If alcohol consumption upregulates CYP2E1 in retinal tissues in the same way that it does in liver [40,41,65], it should be considered as a risk factor since it could aggravate retinal degeneration in those patients with high baseline oxidative stress levels due to their ocular pathology including AMD. On the other hand, because antioxidants such as NAC and DAS are effective against CYP2E1-mediated deleterious effects, their possible role as adjuvant therapies in retinal diseases deserves further research.

## Supplementary Materials

The following are available online at https://www.mdpi.com/2076-3921/9/9/776/s1, Table S1: Mean of pixel density of each protein.
