# Supplementary material for: Ethanol-Induced Oxidative Stress Modifies Inflammation and Angiogenesis Biomarkers in Retinal Pigment Epithelial Cells (ARPE-19): Role of CYP2E1 and its Inhibition by Antioxidants
Source: Antioxidants (Basel). 2020 Aug 21;9(9):776. doi: 10.3390/antiox9090776 (PMC7555214; doi:10.3390/antiox9090776)
Supplement: Supplementary file 1 [file antioxidants-09-00776-s001.pdf]

Supplementary material

Supplementary table 1. Mean of pixel density of each protein.

PROTEIN	EtOH treatment			
	CTL	200 mM	400 mM	600 mM
Activin	0,485	0,498	0,519	0,252
ADAMTS-1	0,069	0,164	0,129	0,104
Angiogenin	0,193	0,350	0,173	0,266
Angiopoietin-1	0,828	0,573	0,633	0,560
Angiopoietin-2	1,566	1,129	1,688	1,051
Angiostatin/Plasminogen	0,277	0,256	0,370	0,381
Amphiregulin	0,319	0,123	0,281	0,251
Artemin	1,113	0,971	0,875	1,016
Coagulation Factor III	27,372	24,126	31,003	24,420
CXCL16	0,292	0,162	0,346	0,246
DPPIV	8,430	8,749	7,631	6,033
EGF	0,215	0,060	0,123	0,057
EG-VEGF	0,605	0,566	0,619	0,666
Endoglin	0,228	0,145	0,197	0,165
Endostatin/Collagen XVIII	1,102	0,734	0,570	0,413
Endothelin-1	0,706	0,553	1,175	0,342
FGF acidic	9,584	7,866	5,791	6,357
FGF basic	35,274	35,383	37,355	32,898
FGF-4	0,146	0,004	0,209	0,247
FGF-7	0,163	0,078	0,012	0,000
GDNF	0,163	0,247	0,306	0,363
GM-CSF	0,077	0,264	0,278	0,146
HB-EGF	1,151	0,938	0,846	0,806
HGF	0,254	0,235	0,128	0,082
IGFBP-1	1,258	1,182	1,459	1,326
IGFBP-2	0,387	0,377	0,480	0,510
IGFBP-3	1,356	1,828	1,128	0,618
IL-1ß	0,261	0,170	0,352	0,348
IL-8	0,241	0,327	0,157	0,113
LAP (TGF-ß1)	1,409	1,280	0,965	0,501
Leptin	0,064	0,118	0,088	0,117
MCP-1	0,146	0,128	0,270	0,187
MIP-1α	0,287	0,258	0,459	0,422
MMP-8	0,226	0,315	0,340	0,626
MMP-9	0,245	0,364	0,579	0,315
NRG1-β1	0,285	0,203	0,285	0,105
Pentraxin 3 (PTX3)	0,216	0,385	0,335	0,487
PD-ECGF	0,445	0,362	0,563	0,589
PDGF-AA	0,210	0,354	0,334	0,298
PDGF-AB/PDGF-BB	0,184	0,162	0,170	0,012
Persephin	1,854	1,613	1,814	1,672

Platelet Factor 4 (PF4)	0,640	0,546	0,597	0,516
PlGF	0,528	0,366	0,506	0,503
Prolactin	0,402	0,332	0,406	0,260
Serpin B5	0,128	0,179	0,027	0,037
Serpin E1	15,487	20,071	15,791	16,872
Serpin F1 (PEDF)	0,277	0,393	0,357	0,220
TIMP-1	3,118	3,021	3,918	6,704
TIMP-4	0,216	0,220	0,376	0,222
Thrombospondin-1	2,300	2,896	2,533	0,911
Thrombospondin-2	0,159	0,071	0,237	0,204
uPA	0,316	0,477	0,578	1,527
Vasohibin	0,266	0,257	0,537	0,300
VEGF	0,744	0,711	0,609	0,481
VEGF-C	0,048	0,117	0,301	0,261

Values are the mean of pixel density of the pair of duplicate spots representing each protein on the immunoblotted membranes of the Proteome Profile Array. Membranes were incubated with ARPE-19 protein samples after EtOH treatment for 24 hours.
